# The Pharmacological Effects and Pharmacokinetics of Active Compounds of *Artemisia capillaris*

**DOI:** 10.3390/biomedicines9101412

**Published:** 2021-10-08

**Authors:** Tun-Pin Hsueh, Wan-Ling Lin, Jeffrey W. Dalley, Tung-Hu Tsai

**Affiliations:** 1 Institute of Traditional Medicine, School of Medicine, National Yang-Ming Chiao-Tung University, Taipei 112, Taiwan; melaopin@gmail.com; 2 School of Traditional Chinese Medicine, Chang Gung University, Taoyuan 333, Taiwan; 3 Department of Chinese Medicine, Kaohsiung Chang Gung Memorial Hospital and Chang Gung University College of Medicine, Kaohsiung 833, Taiwan; 4 Department of Traditional Medicine, Kaohsiung Veterans General Hospital, Kaohsiung 813, Taiwan; slingr23@gmail.com; 5 Institute of Education, National Sun Yat-sen University, Kaohsiung 804, Taiwan; 6 Department of Psychology, University of Cambridge, Cambridge CB2 3EB, UK; jwd20@cam.ac.uk; 7 Department of Psychiatry, University of Cambridge, Cambridge CB2 0SZ, UK; 8 Department of Education and Research, Taipei City Hospital, Taipei 103, Taiwan

**Keywords:** *Artemisia*, pharmacokinetics, pharmacology, herbal medicine, Yin-Chen, scoparone

## Abstract

*Artemisia capillaris* Thunb. (*A.* *capillaris,* Yin-Chen in Chinese) is a traditional medicinal herb with a wide spectrum of pharmacological properties ranging from effects against liver dysfunction to treatments of severe cirrhosis and cancer. We used relevant keywords to search electronic databases, including PubMed, Medline, and Google Scholar, for scientific contributions related to this medicinal herb and the pharmacokinetics of its components. The pharmaceutical effects of *A.* *capillaris* contribute to the treatment not only of viral hepatitis, cirrhosis, and hepatocellular hepatoma, but also metabolic syndrome, psoriasis, and enterovirus in the clinic. The bioactive compounds, including scoparone, capillarisin, scopoletin, and chlorogenic acid, exhibit antioxidant, anti-inflammatory, antisteatotic, antiviral, and antitumor properties, reflecting the pharmacological effects of *A.* *capillaris*. The pharmacokinetics of the main bioactive compounds in *A. capillaris* can achieve a maximum concentration within 1 hour, but only chlorogenic acid has a relatively long half-life. Regarding the use of the *A. capillaris* herb by health professionals to treat various diseases, the dosing schedule of this herb should be carefully considered to maximize therapeutic outcomes while lessening possible side effects.

## 1. Introduction

The original uses of *Artemisia capillaris* Thunb. (*A. capillaris,* Yin-Chen in Chinese) in traditional Chinese medicine included the treatment of pyrexia, jaundice, and dysuria. Current basic studies of traditional Chinese medicine aim to find molecular, cytological and pharmacological evidence supporting the use of traditional Chinese herbal medicines to confirm the nature of active ingredients and to explore the roles of these compounds in the treatment of various diseases. Studies of this traditional herb provide reliable empirical evidence for the development and progress of this traditional Chinese medicine, which has been widely used in the treatment of various diseases ranging from liver inflammation to severe cirrhosis and liver cancer [1,2,3]. Previous studies have shown that the whole plant exerts antioxidant, anti-inflammatory, antisteatotic, antiviral, and antitumor effects [4,5,6,7]. Most of the therapeutic effects can be attributed to the major or minor compounds found in medicinal herbs [8].

However, the bioactive components of an herb are influenced by the plant species and origin. For example, the clinical use of *Artemisia* in traditional Chinese medicine includes *A. capillaris* and *Artemisia scoparia (A. scoparia)* [9]. Though *A. capillaris* is the main species used in medicinal formulations, *A.* *scoparia* contains higher levels of the essential active compound scoparone, whereas chlorogenic acid is abundant in *A.* *capillaris* [9]. The harvested time and region of the plant also affect the chemical compositions of bioactive compounds. Both capillarisin and scoparone (6,7-dimethylesculetin) content reach peak levels in the leaf of *A. capillaris* at the end of July. However, the maximal level of capillarisin and scoparone is detected in the capitulum in early August and early September, respectively [10]. The appropriate time to harvest *A. capillaris* is an important consideration that affects pharmacological actions, including possible toxicity from scoparone. Thus, the therapeutic benefits of *A. capillaris* are influenced by various parts of the plant and harvest time, and should be balanced based on the contents of bioactive components.

The level of bioactive compounds determines an individual’s pharmacokinetic and pharmacodynamic reactions to herbal medicines. Recent studies have invested considerable efforts into exploring the multicomponent pharmacokinetics of herbal medicines and to elucidate relevant metabolic processes. For instance, an herbal formula containing *A. capillaris* was found to be disproportional to its administering dose in the area under the curve (AUC) by monitoring its bioactive components [11], which indicated pharmacokinetic saturation leading to excessive dosage or potential interaction with other drugs. The pharmacokinetic investigation of *A. capillaris* combined herbal formula upon dose appeared to interact with spironolactone, including the urinary sodium-to-potassium ratio [12]. In addition, a lack of information on the therapeutic window and pharmacodynamics that guide pharmacokinetics makes the efficacy and safety of herbal medicine questionable.

Previous studies reported pharmacokinetic properties or pharmacological activity of *A.* *capillaris* aims on one bioactive component or single therapeutic function. Few reviews summarize the pharmacokinetic properties of major bioactive compounds as well as currently proved pharmacological usage as suggestions for future potential applications of the plant. To the best of our knowledge, *A. capillaris* has been known for the treatment of liver disease but awareness is rare for the treatment for metabolic syndrome, psoriasis, or antifibrotic effects that indicate that this plant has an expanded range of therapeutic activities. Thus, the present review of pharmacological effects of *A.* *capillaris* and linking to the pharmacokinetics of extracted bioactive compounds aims to discover additional applications of this herbal medicine.

## 2. Research Methods

Scientific search engines, such as PubMed, Medline, and Google Scholar were used to collect all published articles on ethnomedicinal use, biological properties, and pharmacokinetics of *A.* *capillaris.* A synopsis of this search is presented in this review. Acquired manuscripts were assessed and identified based on the title and abstract. The following search terms were used as keywords: *Artemisia*, *Artemisia capillaris*, Yin-Chen (Chinese name), Yin-Chen-Hao-Tang (an ancient Chinese formula), and pharmacokinetics. The reference lists of retrieved publications were also examined to identify other relevant studies.

## 3. Pharmacological Effects of *Artemisia capillaris*

### 3.1. Viral Hepatitis B Infection

Hepatitis B virus (HBV) infection can progress to liver cirrhosis or liver cancer. At present, the therapeutic options for this disease are limited by agents used for eradication of HBV or of side effects of antiviral therapies. Compounds isolated from *A. capillaris* inhibit the secretion of HBsAg or HBeAg or replication of HBV DNA [13]. HBeAg secretion and HBV DNA replication in HepG cells are significantly inhibited by 90% ethanol extract of *A. capillaris* [14], and this activity is due to chlorogenic acid analogs and enynes found in *A. capillaris* [15].

### 3.2. Cirrhosis and Hepatoprotective Effects

Cirrhosis refers to the late stage of scarring caused by repeated pathological destruction and regeneration of the liver due to various forms of liver diseases. A strategy for the prevention of liver damage involves pretreatment with an aqueous extract of *A. capillaris*, which significantly reduces oxidative stress in the liver induced by 2,2′-azobis(2-amidinopropane) dihydrochloride (AAPH), as demonstrated by a decrease in the level of liver injury based on the enzyme markers aspartate transaminase (AST) and alanine transaminase (ALT) in rats [16]. Herbal formulations consisting of *A. capillaris* and *Alisma canaliculatum* (Saeng-kankunbi-tang, SKT) also prevent tert-butyl hydroperoxide (tBHP)-induced oxidative injury in HepG2 hepatocytes and acute oxidative hepatic damage caused by carbon tetrachloride (CCl_4_) in mice [17].

Antifibrotic effects of *A. capillaris* have also been reported. The β-sitosterol component derived from *A. capillaris* alleviates dimethylnitrosamine (DMN)-induced hepatofibrosis in mice [18]. In a model of bile duct ligation (BDL)-induced cholestatic fibrosis, aqueous extract of *A.* *capillaris* suppresses the expression of fibrogenic factors, including alpha-smooth muscle actin (α-SMA), platelet-derived growth factor (PDGF), and transforming growth factor-beta (TGF-β), and significantly reduces the levels of cholestatic markers malondialdehyde (MDA) in the serum and hydroxyproline in the liver after 2 weeks of treatment in rats [19]. An increase in the AST, ALT, and MDA levels induced by 30% alcohol plus pyrazole is also ameliorated by aqueous extract of *A. capillaris* in a rat model [20]. This hepatoprotective effect is attributed to an enhancement of antioxidant activity, including glutathione peroxidase (GSH-Px), glutathione reductase (GSH-Rd), catalase, and superoxide dismutase (SOD) [20]. However, another study demonstrated that 5-week treatment with aqueous *A.* *capillaris* extract did not alter liver enzymes, including ALT, AST, and alkaline phosphatase (ALP), in a rat model of CCl_4_-induced hepatic fibrosis, although different results were obtained in the case of *Artemisia iwayomogi* [21]. Another study reached a similar conclusion after administration of methanol extract of *A. capillaris* in rats with bile duct ligation; the results indicated that the serum levels of AST, ALT, and ALP and hepatic levels of hydroxyproline were significantly reduced in the *Polygonum aviculare*-treated group but not in the groups treated with *A.* *capillaris* and aqueous biphenyl dimethyl dicarboxylate [22]. Variability of hepatoprotective effects may be due to extraction or cultivation and should be confirmed by assessment of biological components.

### 3.3. Hepatocellular Carcinoma

Hepatocellular carcinoma (HCC) requires effective treatment due to low 10% 5-year survival rate of this disease [23]. Increasing evidence indicates that *A. capillaris* can efficiently suppress the proliferation of human hepatoma cells, and ethanol extract of *A. capillaris* exhibits dose-dependent antiproliferative effects against Huh7 and HepG2 human hepatoma cells mediated by inhibition of cancer cell migration via interleukin-6 (IL-6)-dependent regulation of the signal transducer and activator of transcription 3 (STAT3) pathway [24]. In HepG2 human hepatocarcinoma cells, aqueous extract of *A. capillaris* inhibits nuclear translocation of NF-κB and blocks the degradation of I-κB alpha, leading to inhibition of inflammatory proteins, such as inducible nitric oxide synthase (iNOS), cyclooxygenase-2 (COX-2), and tumor necrosis factor (TNF)-alpha [25]. Additionally, water-soluble macromolecular components of *A. capillaris* dose-dependently inhibit the proliferation of human hepatoma SMMC-7721 cells by inducing the cell-cycle arrest at the G0/G1 phase [26]. Ethyl acetate extract of *A. capillaris* can effectively inhibit the growth and induce apoptosis of hepatocellular carcinoma cells, and these effects are presumed to be mediated by inhibition of angiogenesis via the blockade of the PI3K/AKT/mTOR signaling pathway [27]. Dried leaves of *A. capillaris* use a similar mechanism to induce apoptosis in HepG2 and Huh7 cells and to suppress tumor growth in mouse xenograft models [28].

### 3.4. Metabolic Syndrome and Diabetes

Metabolic syndrome is associated with a fivefold higher risk for type 2 diabetes (T2DM). Overabundance of circulating fatty acids is the major contributor to pathophysiology of metabolic syndrome. Fatty acids inhibit antilipolytic effect of insulin and induce lipolysis of stored triacylglycerol molecules in the adipose tissue, which leads to insulin resistance [29]. Glucosidase inhibitors delay the absorption of carbohydrates to decrease postprandial hyperglycemia. The inhibitory effect of *A. capillaris* on α-glucosidase is the most potent among that of 12 various *Artemisia* species, and this effect is even stronger than inhibition by acarbose [30]. Rats administered a high-fat diet (HFD) were treated with *A. capillaris* extracts for 7 weeks, and the body weight and levels of serum triglycerides (TG), total cholesterol (TC), and low-density lipoprotein cholesterol (LDL-c) of treated rats were measured and found to be lower than those in the HFD-induced obesity control group; however, the levels of high-density lipoprotein cholesterol (HDL-c) were not significantly different between the groups [31]. Similar results were obtained in another study using a traditional *A. capillaris* formula. The treatment abrogated an increase in liver enzymes and lipid parameters, such as TG, TC, and LDL-c, but not HDL-c, in HFD-fed rats. These effects were apparently mediated by miR-122-induced downregulation of fatty acid synthase genes in HepG2 cells [32].

Free fatty acids induce insulin resistance and inflammation in insulin-targeted organs, indicating a major link between obesity, insulin resistance, inflammation, and T2DM development [33]. Free fatty acid-induced steatosis was relieved and nonalcoholic steatohepatitis (NASH)-related mechanisms were inhibited in HepG2 cells treated with 30% ethanolic extract of *A. capillaris*, including activation of c-Jun NH2 terminal kinase (JNK) and p53-upregulated modulator of apoptosis (PUMA) [34]. An n-BuOH fraction of methanol extract of *A. capillaris* contains vicenin 2, which potently inhibits diabetes-targeting enzymes α-glucosidase, protein tyrosine phosphatase 1B (PTP1B), and rat lens aldose reductase (RLAR) [35]. However, *A. capillaris* extract combined with *Alisma rhizome* extract does not alter the levels of lipid metabolites, such as triacylglycerol and diacylglycerol, in diabetic mice [36]. In contrast, *Hericium erinaceus* cultivated with *A. capillaris* can elevate the HDL level and lower atherogenic index and cardiac risk factor values in hyperlipidemic rats; these effects were more pronounced than the effects of currently used drugs simvastatin and atorvastatin [37]. Thus, the regulatory effects of *A. capillaris* on dyslipidemia appear to be influenced by additions of other herbs.

### 3.5. Dermatitis, Psoriasis, and Skin Carcinogenesis

Atopic dermatitis is an inflammatory skin disease caused by an imbalance of Th cells, the overexpression of COX-2 and iNOS, which generate nitric oxide (NO) and prostaglandin E2, respectively, and stimulation of macrophages by lipopolysaccharide. Topical application of *A. capillaris* for 4 weeks reduced the atopic dermatitis scores and plasma levels of histamine and IgE in *Dermatophagoides farinae*-sensitized Nc/Nga mice [38]. Solid fermentation of *Ganoderma lucidum* on *A. capillaris* leaves reduced the expression of endothelial nitric oxide synthase (eNOS) in mice with 2,4-dinitrofluorobenzene (DNFB)-induced atopic dermatitis [39]. *A.* *capillaris* extract cream has been locally applied to skin lesions in a mouse model of imiquimod (IMQ)-induced psoriasis-like disease, and the level of intracellular adhesion molecule-1 (ICAM-1), modified psoriasis area, and severity index (PASI) scores of treated mice were significantly lower than those of mice in other experimental groups [40]. Moreover, the chloroform fraction of methanolic extract of *A. capillaris* markedly decreased the number and incidence of tumors in mice with 12-dimethylbenz(a)anthracene (DMBA)-induced epidermal carcinogenesis compared with the results obtained using other anticarcinogenic medicinal herbs, including *Taxus cuspidata*, *Anthriscus sylvestris*, and *Curcuma longa* [41].

### 3.6. Enterovirus 71 (EV71) and Helicobacter pylori (H. pylori)

Infectious diseases have affected humans for centuries and have civilization-altering consequences. Studies in human foreskin fibroblasts and rhabdomyosarcoma cells demonstrated that aqueous extract of *A. capillaris* dose-dependently protects against EV71 infection, mainly due to inhibition of viral internalization [42]. Acidic polysaccharides from *A. capillaris* are potent inhibitors of adhesion of *H. pylori* to erythrocytes; however, this effect was less potent than that of acidic polysaccharides from *Panax ginseng* (*P. ginseng*) [43]. Aqueous extract of *A. capillaris* has no acid-neutralizing activity and does not prevent histamine secretion from HMC-1 mast cells; however, pretreatment with this aqueous extract decreases HCl/ethanol-induced gastric mucosal lesions [44,45]. A summary of the therapeutic effects of *A. capillaris* is provided in Table 1.

## 4. Pharmacokinetics of Bioactive Compounds Found in *Artemisia capillaris*

**Figure 1:**
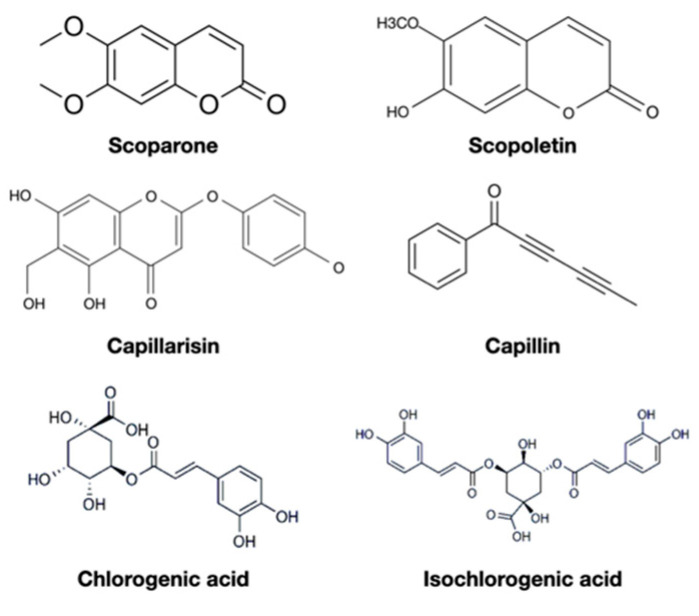
The chemical structure of commonly identified constituents in *Artemisia capillaris*.

*A. capillaris* have been intensively studied to evaluate its effects on healthcare, demonstrating that *A.* *capillaris* has protective effects against hepatitis, cirrhosis, cancer, metabolic syndrome, dermatitis, and microbes, which triggered additional scientific inquiries. Numerous bioactive compounds were therefore extracted and found to have beneficial effects, including anti-inflammatory, antioxidant, antitumor, or even anti-HIV activities. The most commonly identified constituents include scoparone, capillarisin, and chlorogenic acid (Table 2) [46,47,48,49]. Their chemical structures are shown in Figure 1. From a practical perspective, these bioactive compounds composed of the pharmacology of *A. capillaris* need rigorous pharmacokinetics research to provide science-based dosage recommendations for various therapeutic properties of *A. capillaris*. Realizing the pharmacokinetics of bioactive compounds in *A. capillaris* could improve the administration schedule to achieve medicinal effects in an efficient manner. The pharmacokinetics of bioactive compounds in *A. capillaris* are discussed below.

### 4.1. Scoparone

Scoparone is considered the main and most important active constituent of *A. capillaris*. Analysis of Soxhlet extracts of *A. capillaris* indicated that the plant contains higher levels of scoparone than the levels of capillarisin or chlorogenic acid [46]. This compound was considered of interest due to its preventive and therapeutic effects against liver disease, which prompted subsequent investigations [50,51]. Scoparone isolated from *A. capillaris* has been shown to have antioxidant properties demonstrated by a reduction in the MDA and ALT levels in cold-preserved rat hepatocytes [52]. Scoparone decreases the levels of interleukin (IL)-1-beta, IL-6, and TNF-alpha due to anti-inflammatory activities of the compound and suppresses the levels of iNOS and COX-2 in IFN-gamma- or LPS-stimulated cells [53]. Moreover, scoparone inhibits the transcriptional activity of peroxisome proliferator-activated receptor gamma (PPARγ) and downregulation of the target genes, and this effect leads to inhibition of triglyceride (TG) accumulation in mature adipocytes [54]. Choleretic effect of scoparone is indirectly potentiated by cytochrome P4501A2 via the bile salt export pump promoter [55]. Scoparone is considered a potential hepatoprotective candidate for hepatitis therapy based on published evidence [56]. Additionally, scoparone 60 mg/kg daily could alleviate angiotensin II infusion-induced cardiac hypertrophy and fibrosis in mice with maintaining cardiac output, left ventricular pressure, and left ventricular workload [57,58].

Analysis of pharmacokinetic parameters of scoparone could achieve a wide range of the C_max_ values from 0.02 mg/L or up to 16.1 mg/L accompanying with standard compounds as Yin-Chen-Hao-Tang ingredients, and most T_max_ values ranged from 6 to 54 min with the exception of one study that reported a T_max_ value of 1.9 h after combined administration with *Gardenia jasminoides* Ellis [59]; the corresponding elimination half-life ranges from 25.8 min to 5.11 h [11,59,60,61,62,63]. The IC_50_ value for scoparone-associated significant inhibition of the proliferation of DU145 prostate cancer cells is 8.5 mg/L (41.3 μmol/L) [64]. High levels of scoparone in *A. capillaris* are apparently responsible for inhibition of prostate cancer proliferation. Consequently, two characteristics of scoparone have been noticed. First, a combination of geniposide delay absorption of scoparone and combined with geniposide and rhein increased the AUC [63]. Additionally, the herbal formula Yin-Chen-Hao-Tang has been used to illustrate nonlinear pharmacokinetic properties of scoparone, which represents a potential enhancement of pharmacological effects of this drug [11].

### 4.2. Scopoletin

Scopoletin isolated from *A. capillaris* contributes to bile secretion similarly to scoparone but has no effect on bile acid and cholesterol secretion [48]. These effects may be due to inhibition of lipid biosynthesis, which results in downregulation of gene expression related to cholesterol, triglyceride synthesis, and inflammation induced by steatosis [65]. This compound may assist in lowering postprandial hyperglycemia and improving antidiabetic treatments [66]. Scopoletin can enhance histone deacetylase expression to inactive p53 in human lung fibroblasts, which leads to autophagy-related antiaging effects [67]. Moreover, scopoletin is cytotoxic toward cancer cells, such as prostate cancer cells (PC-3) and acute lymphoblastic leukemia cells [68,69]. Although NF-κB activation by scopoletin implies a resistance mechanism of cancer cells, the main resistance mechanisms, such as ATP-binding cassette (ABC) transporters, EGFR, and TP53, do not affect cellular resistance to scopoletin [70].

Oral administration of pure scopoletin at the doses of 5, 10, or 20 mg/kg results in the C_max_ values in the plasma of 49.8, 101.3, or 217.3 μg/L, respectively, reached within 0.4 h [71]. Another study of oral administration at a dose of 50 mg/kg resulted in the C_max_ value of 0.4 μg/L within 14 min. However, treatment with a decoction prepared from 720 g of *A. capillaris* resulted in a relatively low plasma concentration of only 3.5 μg/L after feeding [61]. Similarly, administration of *Radix angelicae pubescentis* extract containing 0.055 mg/kg scopoletin did not result in detectable levels of the compound in rat plasma [72]. It has also been revealed with a short elimination half-life in dogs [73]; thus, scopoletin is not easily absorbed or rapidly metabolized when administered in an herbal formula. The IC_50_ value of scopoletin for human CCRF-CEM leukemia cells is 499.6 μg/L [69]. These findings indicate that the level of scopoletin in *A. capillaris* and *Radix angelicae pubescentis* is insufficient to detect pharmacological effects of pure scopoletin against leukemia cells in the clinic.

### 4.3. Capillarisin

Capillarisin is derived naturally from chromone. The compound has antioxidant, anti-inflammatory, and potential antitumor properties [74], and antioxidant and anti-inflammatory activities are regulated via the Nrf2/ARE-dependent pathway and activation of ERK, JNK, NF-κB, and MAPK [75,76,77]. Capillarisin also has inhibitory effects on prostate carcinoma cells, apparently mediated by suppression of the activation of androgen receptor, survivin, matrix metalloproteinase (MMP)-2, MMP-9, and STAT3 [78,79].

Treatment with Yin-Chen-Hao-Tang (YCHT) formula, which combines 18 g of *A. capillaris* with *Gardenia jasminoides* Ellis (9 or 12 g) and *Rheum palmatum* L. (6 g), reported two remarkable maximal concentrations of capillarisin of 196 μg/L or 490 μg/L [80,81]. The T_max_ value for capillarisin administered as a component of the YCHT decoction ranges from 5 to 39 min, and the AUC is highly variable, with elimination half-lives ranging from 26 to 159 min. Capillarisin has notably higher affinity to human serum albumin than that of scoparone, indicating that capillarisin bioactivity has a stationary phase [82]. Capillarisin (95% (wt)) has inhibitory effects on human hepatoma Hep-G2 and HUH7 cells, with the IC_50_ values of 72 and 105 μg/mL, respectively [46], and the IC_50_ values for inhibition of the migration and proliferation of colon cancer cells are 92.1 and 76.7 µg/mL, respectively [83]. 

### 4.4. Capillin

Capillin extracted from *Artemisia capillaris* spica inhibits apoptosis induced by transforming growth factor-beta 1 (TGF-β1), which is observed in various inflammatory liver diseases [84]. Human leukemia HL-60 cells are induced to undergo apoptosis after treatment with capillin, and this effect is regulated by activation of the JNK pathway [85]. Antitumor effects of capillin have also been detected in colon carcinoma (HT29 cells), pancreatic carcinoma (MIA PaCa-2 cells), epidermoid carcinoma of the larynx (HEP-2 cells), and lung carcinoma (A549 cells) [84]. Moreover, capillin is a potent inhibitor of α-glucosidase, protein PTP1B, and RLAR for management of diabetes and related complications [86]. However, extraction of capillin from *A. capillaris* has been rarely reported; thus, the pharmacokinetics of capillin are unknown. Further pharmacokinetic studies based on the IC_50_ values should be considered in the investigation of antitumor properties of the compound.

### 4.5. Chlorogenic Acid

Chlorogenic acid is the most abundant compound (38.5 mg/g) in hydroethanolic extract of *A. capillaris* [87]. Chlorogenic acid is also extensively enriched in various foods, such as coffee, tea, cocoa, citrus fruits, berry fruits, apples, and pears [88]. The biological activities of chlorogenic acid have been reported against various diseases, including metabolic syndrome, hypertension, and diabetes [89,90,91], indicating a broad range of anti-inflammatory, antihyperglycemic, and antioxidant activities of the compound [92,93,94]. The mechanism of action of chlorogenic acid mainly involves uptake and synthesis of fatty acids in the liver by modulating hepatic peroxisome proliferator-activated receptor γ or liver X receptors-α (PPARαγ, LXRα) [95]. The compound suppresses inflammation induced by a high-fat diet due to scavenging of reactive oxygen species (ROS) [96]. Chlorogenic acid regulates apoptosis-related genes, and this effect may contribute to the anticancer effect in a lung cancer cell line [97].

Administration of YCHT results in a C_max_ for chlorogenic acid of 33 μg/L or 78 μg/L within 27 min, but the half-life can be minutes to several days [80,81]. Administration of 60 mg/kg dose of chlorogenic acid in extracts *Lonicerae japonicae* Flos achieved a maximum concentration of 2.4 mg/L in the plasma of beagle dogs after 1.0 h [98]. *Eucommia ulmoides* extract containing 1.2, 2.6, and 5.1 mg/kg chlorogenic acid acquired C_max_ of 22.5, 39.8, and 61.0 ng/mL, respectively, within 26 min for all three concentrations after its administration to rats [99]. Chlorogenic acid has been administered to humans by 10 commercially available products with an average content of 3.6 mg, which achieved a maximal concentration of 0.76 ng/mL after approximately 1 h [100]. Though chlorogenic acid was found to be not well-absorbed from the digestive tract [101], the currently available pharmacokinetics of chlorogenic acid in *A. capillaris* revealed rapid time to maximum concentration with up to a month of half-life. Future studies in pharmacology of *A. capillaris* should consider the prolonged elimination in the pharmacokinetics of chlorogenic acid.

### 4.6. Isochlorogenic Acid

Other constituents isolated from *Artemisia capillaris* include isoscopoletin, artepillin, esculetin, isochlorogenic acid, β-sitosterol, and quercetin. Natural isochlorogenic acid refers to a mixture of several isomers. Isochlorogenic acid A is the most or second-most abundant component found in ethanol extract of *A. capillaris* [38,87], and is also a major bioactive constituent in other medicinal plants such as *Lonicerae japonicae* Flos, *Gynura divaricata*, and *Laggera alata* [102,103,104]. Isochlorogenic acid A probably blocks HBV replication to provide for potent anti-HBV activity and induces heme oxygenase-1 (HO-1) expression, which leads to antioxidant effects [104]. The viral load of enterovirus 71 and cytokine secretion decreased after treatment with isochlorogenic acid C [105]. Antioxidant effects and suppression of profibrogenic factors by isochlorogenic acid B are mediated via the Nrf2 and miR-122/HIF-1α signaling pathways to protect against fibrosis in nonalcoholic steatohepatitis (NASH) [106].

The pharmacokinetics of isochlorogenic acid from *A. capillaris* extracts have not been reported. Analysis of pharmacokinetics of isochlorogenic acid after administration of 5.0 g/kg of *Erigeron breviscapus* ethanol extract, which contains 16.56 mg/kg isochlorogenic acid A and 10.64 mg/kg isochlorogenic acid C, revealed the C_max_ values of 1.033 μg/mL and 0.230 μg/mL after 1.17 and 0.11 h, respectively [107]. Oral administration of isochlorogenic acid C at the doses of 5 mg/kg or 25 mg/kg resulted in the T_max_ values ranging from 30 min to 1 h [108]. However, the bioavailability of isochlorogenic acid C is relatively low, ranging from 14.4% to 16.9%. Although the compound has a linear pharmacokinetics profile, poor gastrointestinal absorption and low potency of the compound in herbal medicines may diminish potential therapeutic applications of isochlorogenic acid C.

**Figure 2:**
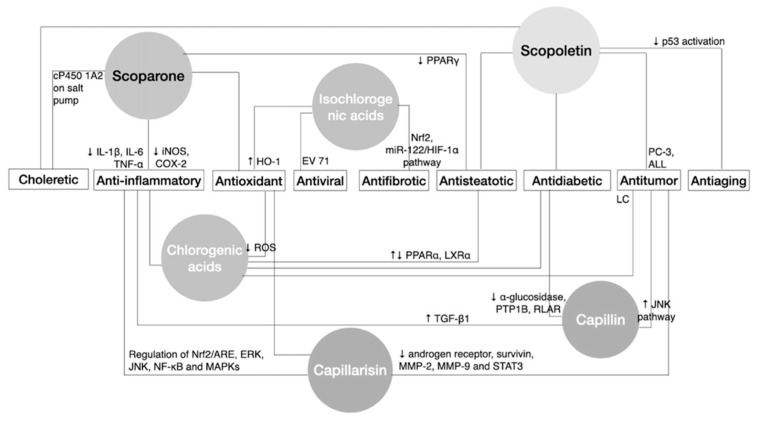
Schematic illustration of the bioactive components of *Artemisia capillaris* contributing to its therapeutic effects. Bioactive compounds include scoparone, scopoletin, capillarisin, capillin, chlorogenic acid, and isochlorogenic acid, which induce pharmacological effects of *A. capillaris* in a synergistic manner. PC-3: prostate cancer cells; ALL: acute lymphoblastic leukemia cells; LC: lung cancer cells; ↓ decrease or inhibit effects; ↑ increase or enhance effects; ↓↑ modulate effects.

**Figure 3:**
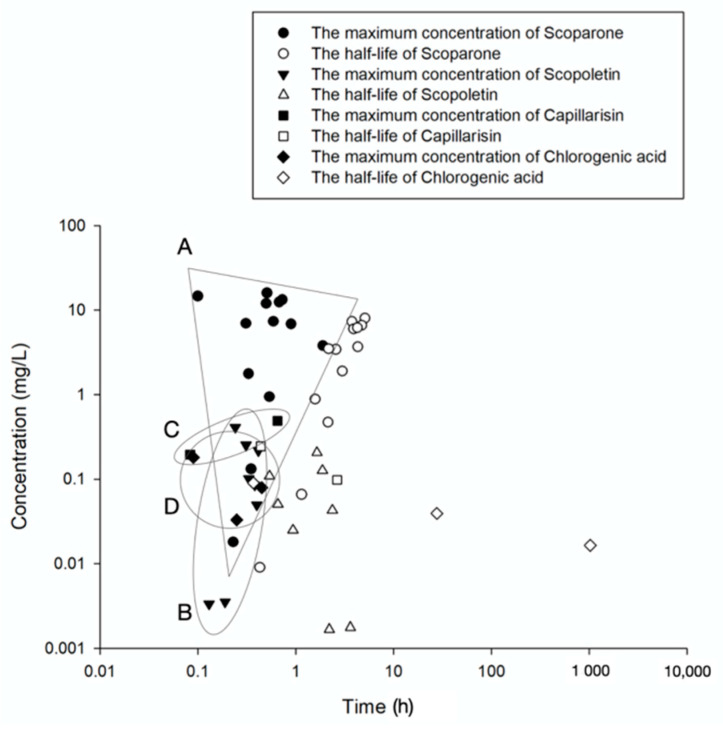
The pharmacokinetic extent of bioactive compounds in *Artemisia capillaris*. The figure illustrates the absorption and elimination of scoparone, scopoletin, capillarisin, and chlorogenic acid in vivo after oral administration of *Artemisia capillaris* extracts. The black symbols represent the maximum concentration (C_max_) of the main bioactive compounds, including scoparone, scopoletin, capillarisin, and chlorogenic acid. The white symbols represent the half-life (t_1/2_) of these compounds. A, B, C, D indicate the area of the C_max_ of scoparone, scopoletin, capillarisin, and chlorogenic acid, respectively.

Analysis of the pharmacodynamic properties of bioactive components of *A.* *capillaris*, such as the choleretic, anti-inflammatory, and antioxidant effects, are mainly from scoparone, scopoletin, capillarisin, capillin, and chlorogenic acids, while antidiabetic, antisteatotic, and antitumor effects are from scopolectin, capillarisin, capillin, and chlorogenic acids (Figure 2). Considering the relatively low plasma concentration detected from scopoletin, the antitumor properties of *A. capillaris* consequently in part come from capillarisin, capillin, and chlorogenic acids. Despite the unknown pharmacokinetics of capillin, all compounds could reach the corresponding C_max_ within an hour and diminished to half-concentration within 5 hours except for chlorogenic acids, which could last more than a month (Figure 3).

## 5. Conclusions

*A. capillaris* has a wide spectrum of pharmacological properties. In addition to HBeAg inhibition, lowering of AST and ALT, and induction of apoptosis of HCC cells, it also lowers TG, TC, and LDL, ICAM-1, inhibits α-glucosidase, and causes viral internalization and *H.* *pylori* adhesion. Published studies have revealed these effects were attributed to verified bioactive compounds such as scoparone, scopoletin, capillarisin, capillin, and chlorogenic acids. These commonly extracted bioactive compounds induce pharmacological effects, including anti-inflammatory, antioxidant, choleretic, antisteatotic, antidiabetic, and antitumor activities, and synergistically contribute to the therapeutic effects of *A. capillaris*. Considering the pharmacokinetics of main bioactive compounds in *A. capillaris*, the choleretic, anti-inflammatory, and antioxidant effects that partly come from scoparone and scopoletin contributing to anti-hepatitis treatment could achieve a therapeutic window within a relatively short time. On the other hand, for antisteatotic, antidiabetic, and antitumor activities attributed to capillarisin, capillin, and chlorogenic acids with relatively low C_max_ and a long half-life, the dosing may need to be adjusted to suit a particular therapeutic purpose. Pharmacokinetic parameters of some bioactive compounds in *A. capillaris,* including scoparone and capillarisin, were acquired from the herbal formula Yin-Chen-Hao-Tang, which significantly altered the pharmacokinetics of bioactive compounds. Regarding the use of this herb by health professionals for treatments of various diseases, the development of *A. capillaris* in composition or dosing schedule for more effective and efficient treatment clinically should be considered for future clinical studies in patients.

A worldwide outbreak of respiratory infection beginning in December 2019 has challenged scientists to find antiviral agents for the treatment of the disease. Researchers proposed the development of *Artemisia annua* for the treatment of COVID-19 due to its antiviral and antioxidant activity in pulmonary fibrosis [109]. *A. capillaris* belongs to the same genus as *A. annua*, has antioxidant, antiviral, and antifibrotic activities, and contains the same bioactive compounds, including scoparone and scopoletin [110,111]. Moreover, *A. capillaris* has been successfully applied in a formula as a complementary treatment for patients with pulmonary fibrosis at critical illness and recovery stages in Taiwan [112]. The present review describes the pharmaceutical properties and pharmacokinetics of bioactive compounds of *A.* *capillaris*. Future studies of *A. capillaris,* including verifying the pharmacokinetics of capillin and isochlorogenic acid, which have antioxidant, antiviral, and antitumor effects, alongside considerations of harvest time and different parts of the plant, are needed to inform the effective doses of A. capillaris for clinical applications.

## Figures and Tables

**Table 1:** Experimental therapeutic effects of *Artemisia capillaris*.

Disease	Extract	Doses/Concentrations	Time Period	Results/Mechanism	Active Compounds	Reference
Hepatitis B	90% ethanol	76.1 μg/mL	NR	inhibition of HBeAg secretion and HBV DNA replication in HepG cells	chlorogenic acid	[14]
Cirrhosis	water	10 mg/kg	NR	alleviation of hepatic fibrosis in a DMN-induced mouse model	β-sitosterol	[18]
	water	25 or 50 mg/kg	2 weeks	suppression of fibrogenic factors, including hepatic α-SMA, PDGF, and TGF-β	NR	[19]
	water	7.5 g/kg	7 days	lowering of AST and ALT in a rat model of AAPH liver damage	NR	[16]
	water	50 or 100 mg/kg	10 days	amelioration of AST, ALT, and MDA in an alcohol-pyrazole-fed rat model; an increase in antioxidant activity due to GSH-Px, GSH-Rd, catalase, and SOD	NR	[20]
	water	25 or 50 mg/kg	6 weeks	unchanged ALT, AST, and ALP in a rat model of CCl_4_-induced liver fibrosis	NR	[21]
Hepatocellular carcinoma	70% ethanol	100 and 250 μg/mL	NR	inhibition of IL-6 mediated STAT3 pathway in Huh7 and HepG2 human hepatoma cells	NR	[24]
	water	50, 150, and 300 mg/kg	NR	Inhibition of NF-kappaB translocation, inhibition of iNOS, COX-2, and TNF-alpha in HepG2 cells	NR	[25]
	water	25–200 µg/mL	NR	cell cycle arrest at the G0/G1 phase in SMMC-7721 cells	NR	[26]
	70% ethanol	50 and 100 μg/ml	NR	Induction of apoptosis of HCC cells by blocking the PI3K/AKT/mTOR signaling pathway	NR	[27]
	70% ethanol	100 μg/mL	15 days	induction of apoptosis in HepG2 and Huh7 cell; suppression of tumor growth in a mouse xenograft	NR	[28]
Metabolic syndrome and diabetes	methanol	0.4% and 0.8% extract	7 weeks	lowering of TG, TC, and LDL-c levels in high-fat diet-fed rats; a decrease in fatty acid synthase expression via miR-122	NR	[31,32]
	30% ethanol	100 μg/mL	NR	inhibition of activation of JNK and PUMA in NASH	NR	[34]
	methanol	IC50 values: 270.5 μM (α-glucosidase); 7.85 μM (RLAR); 139.75 μM (PLAR)	NR	inhibition of α-glucosidase, PTP1B, and RLAR.	vicenin 2	[35]
Dermatitis, psoriasis, and skin carcinogenesis	70% ethanol	10 mg	4 weeks	reduction in atopic dermatitis scores, histamine, and IgE in *Dermatophagoides farinae*-sensitized Nc/Nga mice (topical).	chlorogenic acid, caffeic acid, isochlorogenic acid A, hyperoside, isoquercitrin, and scoparone	[38]
	70% ethanol	250 mg/kg	5 days	lowering of ICAM-1 and modified PASI scores in a mouse model of induced psoriasis-like disease (topical)	chlorogenic acid, 3,5-dicaffeoylquinic acid, 4,5-dicaffeoyl-quinic acid, and scoparone	[40]
	methanol	50 g sample in 500 mL of MeOH	7 days	reduction in the tumor number and incidence in a mouse mode of DMBA-induced epidermal carcinogenesis	camphor, 1-borneol, coumarin, and achillin	[41]
Enterovirus 71 and *H. pylori* l	water	10 μg/mL	NR	effect against EV71 infection due to inhibition of viral internalization	chlorogenic acid	[42]
	water	0.2–2.8 mg/mL	NR	inhibition of *H. pylori* adhesion to erythrocytes	acidic polysaccharide	[43]
	water	200 or 400 mg/kg	NR	inhibition of lipid peroxide formation due to SOD activation, a reduction in proinflammatory cytokines IL-6 and IL-1β mediated by NF-κB downregulation	NR	[44]
	ethanol	30 or 100 mg/kg	1 h prior	attenuation of ethanol-induced proinflammatory cytokines IL-1β and interferon-γ and ICAM-1	NR	[45]

α-SMA, alpha-smooth muscle actin; AAPH, 2,2′-azobis(2-amidinopropane) dihydrochloride; ALT, alanine transaminase; ALP, alkaline phosphatase; AST, aspartate transaminase; CCl_4_, carbon tetrachloride; COX-2, cyclooxygenase-2; GSH-Px, glutathione peroxidase; GSH-Rd, glutathione reductase; ICAM-1, intracellular adhesion molecule-1; IL-1β, interleukin-1 beta; IL-6, interleukin-6; iNOS, inducible nitric oxide synthase; LDL-c, low-density lipoprotein cholesterol; JNK, c-Jun NH2-terminal kinase; MDA, malondialdehyde; NASH, nonalcoholic steatohepatitis; NF-κB, nuclear factor kappa B; NR, not reported; PASI, psoriasis area and severity index; PDGF, platelet-derived growth factor; PTP1B, protein tyrosine phosphatase 1B; PUMA, p53-upregulated modulator of apoptosis; RLAR, rat lens aldose reductase; SOD, superoxide dismutase; STAT3, signal transducer and activator of transcription 3; TC, total cholesterol; TG, triglycerides; TGF-β, transforming growth factor beta; TNF-alpha, tumor necrosis factor alpha.

**Table 2:** Bioactive compounds of *Artemisia capillaris* extraction studies.

Constituents	Extract Solution	Molecular Weight (g/mol)	LC-Mass Detection Ions		References
			[M − H]^−^	[M + H] ^+^	
Scoparone ^1^	supercritical carbon dioxide with ethyl acetate	206.20		207	[46,47,48,49]
Capillarisin ^1^		316.27	315 (M−)	317 (M+)	
Chlorogenic acid ^2^		354.31	191	163	[46,47,49]
Scopoletin ^2^	ethyl acetate and water	192.17	104	234 ^3^	[47,48,49]
Capillin		168.20		140 ^3^	[48,49]
Isoscopoletin		192.17	176 ^4^	133	
Capillene		154.21		153 ^3^	
Capillartemisin B		316.40			
Cirsimaritin		314.29		315	
Capillarisin-7-methyl ether		330.34			[48]
artepillin A		316.40			
Artepillin C		300.40			
Capillarin		198.22			
Eugenol	sodium dodecyl sulfate and sodium borate buffer	164.20		165	[47]
Caffeic acid		180.16	135	163	
Phenol		94.11		95	
*o*-cresol		108.14		108 (M+)	
*m*-cresol		108.14		109 (M+)	
*p*-cresol		108.14		93.0 (M+)	
2-ethylphenol		122.17		107 ^3^	
4-ethylphenol		122.17		107 ^3^	
Artemisidin A	methanol	642.50			[49]
Artemicapins A		250.20			
Artemicapins B		236.18			
Artemicapins C		206.15			
Artemicapins D		440.4			
other 70 known compounds					

^1^ Compounds were found in all references. ^2^ Compounds were found in three references. ^3^ Ions were detected by GC-MS. ^4^ Ions were detected by MS–MS.

## Data Availability

Not applicable.

## References

[1] Tsai T.Y., Livneh H., Hung T.H., Lin I.H., Lu M.C., Yeh C.C. (2017). Associations between prescribed chinese herbal medicine and risk of hepatocellular carcinoma in patients with chronic hepatitis b: A nationwide population-based cohort study. BMJ Open.

[2] Ting C.T., Kuo C.J., Hu H.Y., Lee Y.L., Tsai T.H. (2017). Prescription frequency and patterns of chinese herbal medicine for liver cancer patients in taiwan: A cross-sectional analysis of the national health insurance research database. BMC Complement. Altern. Med..

[3] Liu C.Y., Chu J.Y., Chiang J.H., Yen H.R., Hsu C.H. (2016). Utilization and prescription patterns of traditional chinese medicine for patients with hepatitis c in taiwan: A population-based study. BMC Complement. Altern. Med..

[4] Lee T.Y., Chang H.H., Lo W.C., Lin H.C. (2010). Alleviation of hepatic oxidative stress by chinese herbal medicine yin-chen-hao-tang in obese mice with steatosis. Int. J. Mol. Med..

[5] Lee T.Y., Chang H.H., Kuo J.J., Shen J.J. (2009). Changes of hepatic proteome in bile duct ligated rats with hepatic fibrosis following treatment with yin-chen-hao-tang. Int. J. Mol. Med..

[6] Lee T.Y., Chang H.H., Wu M.Y., Lin H.C. (2007). Yin-chen-hao-tang ameliorates obstruction-induced hepatic apoptosis in rats. J. Pharm. Pharmacol..

[7] Lee T.Y., Chang H.H., Chen J.H., Hsueh M.L., Kuo J.J. (2007). Herb medicine yin-chen-hao-tang ameliorates hepatic fibrosis in bile duct ligation rats. J. Ethnopharmacol..

[8] Wang X., Sun W., Sun H., Lv H., Wu Z., Wang P., Liu L., Cao H. (2008). Analysis of the constituents in the rat plasma after oral administration of yin chen hao tang by uplc/q-tof-ms/ms. J. Pharm. Biomed. Anal..

[9] Tang W., Eisenbrand G. (1992). *Artemisia scoparia* waldst. et kit. and *A. capillaris* thunb. Chinese Drugs of Plant Origin.

[10] Ju I.-O., You D.-H., Song Y.-E., Jang I., Ryu J., Choi S.-R. (2007). Changes of major components and growth characteristics according to harvesting times of artemisia capillaris thunberg. Korean J. Med. Crop. Sci..

[11] Hsueh T.-P., Tsai T.-H. (2018). Preclinical pharmacokinetics of scoparone, geniposide and rhein in an herbal medicine using a validated lc-ms/ms method. Molecules.

[12] Hsueh T.P., Tsai T.H. (2020). Preclinical study of simultaneous pharmacokinetic and pharmacodynamic herb-drug interactions between yin-chen-hao-tang and spironolactone. BMC Complement. Med. Ther..

[13] Zhao Y., Geng C.A., Sun C.L., Ma Y.B., Huang X.Y., Cao T.W., He K., Wang H., Zhang X.M., Chen J.J. (2014). Polyacetylenes and anti-hepatitis b virus active constituents from artemisia capillaris. Fitoterapia.

[14] Zhao Y., Geng C.A., Ma Y.B., Huang X.Y., Chen H., Cao T.W., He K., Wang H., Zhang X.M., Chen J.J. (2014). Uflc/ms-it-tof guided isolation of anti-hbv active chlorogenic acid analogues from artemisia capillaris as a traditional chinese herb for the treatment of hepatitis. J. Ethnopharmacol..

[15] Geng C.A., Yang T.H., Huang X.Y., Yang J., Ma Y.B., Li T.Z., Zhang X.M., Chen J.J. (2018). Anti-hepatitis b virus effects of the traditional chinese herb artemisia capillaris and its active enynes. J. Ethnopharmacol..

[16] Han K.H., Jeon Y.J., Athukorala Y., Choi K.D., Kim C.J., Cho J.K., Sekikawa M., Fukushima M., Lee C.H. (2006). A water extract of artemisia capillaris prevents 2,2′-azobis(2-amidinopropane) dihydrochloride-induced liver damage in rats. J. Med. Food.

[17] Lee I.W., Choi H.Y., Lee J.H., Park S.D., Kim S.M., Ku S.K., Zhao R.J., Kim S.C., Kim Y.W., Choi H.S. (2016). Saeng-kankunbi-tang protects liver against oxidative damage through activation of erk/nrf2 pathway. Chin. J. Integr. Med..

[18] Kim K.S., Yang H.J., Lee J.Y., Na Y.C., Kwon S.Y., Kim Y.C., Lee J.H., Jang H.J. (2014). Effects of beta-sitosterol derived from artemisia capillaris on the activated human hepatic stellate cells and dimethylnitrosamine-induced mouse liver fibrosis. BMC Complement. Altern. Med..

[19] Han J.M., Kim H.G., Choi M.K., Lee J.S., Lee J.S., Wang J.H., Park H.J., Son S.W., Hwang S.Y., Son C.G. (2013). Artemisia capillaris extract protects against bile duct ligation-induced liver fibrosis in rats. Exp. Toxicol. Pathol..

[20] Choi M.K., Han J.M., Kim H.G., Lee J.S., Lee J.S., Wang J.H., Son S.W., Park H.J., Son C.G. (2013). Aqueous extract of artemisia capillaris exerts hepatoprotective action in alcohol-pyrazole-fed rat model. J. Ethnopharmacol..

[21] Wang J.H., Choi M.K., Shin J.W., Hwang S.Y., Son C.G. (2012). Antifibrotic effects of artemisia capillaris and artemisia iwayomogi in a carbon tetrachloride-induced chronic hepatic fibrosis animal model. J. Ethnopharmacol..

[22] Nan J.X., Park E.J., Kim H.J., Ko G., Sohn D.H. (2000). Antifibrotic effects of the methanol extract of polygonum aviculare in fibrotic rats induced by bile duct ligation and scission. Biol. Pharm. Bull..

[23] Altekruse S.F., McGlynn K.A., Reichman M.E. (2009). Hepatocellular carcinoma incidence, mortality, and survival trends in the United States from 1975 to 2005. J. Clin. Oncol..

[24] Jang E., Kim S.Y., Lee N.R., Yi C.M., Hong D.R., Lee W.S., Kim J.H., Lee K.T., Kim B.J., Lee J.H. (2017). Evaluation of antitumor activity of artemisia capillaris extract against hepatocellular carcinoma through the inhibition of il-6/stat3 signaling axis. Oncol. Rep..

[25] Hong S.H., Seo S.H., Lee J.H., Choi B.T. (2004). The aqueous extract from artemisia capillaris thunb. Inhibits lipopolysaccharide-induced inflammatory response through preventing nf-kappab activation in human hepatoma cell line and rat liver. Int. J. Mol. Med..

[26] Hu Y.Q., Tan R.X., Chu M.Y., Zhou J. (2000). Apoptosis in human hepatoma cell line smmc-7721 induced by water-soluble macromolecular components of artemisia capillaris thunberg. Jpn. J. Cancer Res..

[27] Jung K.H., Rumman M., Yan H., Cheon M.J., Choi J.G., Jin X., Park S., Oh M.S., Hong S.S. (2018). An ethyl acetate fraction of artemisia capillaris (ace-63) induced apoptosis and anti-angiogenesis via inhibition of pi3k/akt signaling in hepatocellular carcinoma. Phytother. Res..

[28] Kim J., Jung K.H., Yan H.H., Cheon M.J., Kang S., Jin X., Park S., Oh M.S., Hong S.S. (2018). Artemisia capillaris leaves inhibit cell proliferation and induce apoptosis in hepatocellular carcinoma. BMC Complement. Altern. Med..

[29] Eckel R.H., Grundy S.M., Zimmet P.Z. (2005). The metabolic syndrome. Lancet.

[30] Nurul Islam M., Jung H.A., Sohn H.S., Kim H.M., Choi J.S. (2013). Potent alpha-glucosidase and protein tyrosine phosphatase 1b inhibitors from artemisia capillaris. Arch. Pharm. Res..

[31] Lim D.W., Kim Y.T., Jang Y.J., Kim Y.E., Han D. (2013). Anti-obesity effect of artemisia capillaris extracts in high-fat diet-induced obese rats. Molecules.

[32] Liu L., Zhao J., Li Y., Wan Y., Lin J., Shen A., Xu W., Li H., Zhang Y., Xu J. (2016). Artemisia capillaris formula inhibits hepatic steatosis via an mir122 induced decrease in fatty acid synthase expression in vivo and in vitro. Mol. Med. Rep..

[33] Boden G. (2008). Obesity and free fatty acids. Endocrinol. Metab. Clin. N. Am..

[34] Jang E., Shin M.H., Kim K.S., Kim Y., Na Y.C., Woo H.J., Kim Y., Lee J.H., Jang H.J. (2014). Anti-lipoapoptotic effect of artemisia capillaris extract on free fatty acids-induced hepg2 cells. BMC Complement. Altern. Med..

[35] Islam M.N., Ishita I.J., Jung H.A., Choi J.S. (2014). Vicenin 2 isolated from artemisia capillaris exhibited potent anti-glycation properties. Food Chem. Toxicol..

[36] Kim Y., Lee I.S., Kim K.H., Park J., Lee J.H., Bang E., Jang H.J., Na Y.C. (2016). Metabolic profiling of liver tissue in diabetic mice treated with artemisia capillaris and alisma rhizome using lc-ms and ce-ms. Am. J. Chin. Med..

[37] Choi W.S., Kim Y.S., Park B.S., Kim J.E., Lee S.E. (2013). Hypolipidaemic effect of hericium erinaceum grown in artemisia capillaris on obese rats. Mycobiology.

[38] Ha H., Lee H., Seo C.S., Lim H.-S., Lee J.K., Lee M.-Y., Shin H. (2014). Artemisia capillaris inhibits atopic dermatitis-like skin lesions in dermatophagoides farinae-sensitized nc/nga mice. BMC Complement. Altern. Med..

[39] Son H.U., Lee S., Heo J.C., Lee S.H. (2017). The solid-state fermentation of artemisia capillaris leaves with ganoderma lucidum enhances the anti-inflammatory effects in a model of atopic dermatitis. Int. J. Mol. Med..

[40] Lee S.Y., Nam S., Kim S., Koo J.S., Hong I.K., Kim H., Han S., Kang M., Yang H., Cho H.J. (2018). Therapeutic efficacies of artemisia capillaris extract cream formulation in imiquimod-induced psoriasis models. Evid. Based Complement. Alternat. Med..

[41] Kim Y.S., Bahn K.N., Hah C.K., Gang H.I., Ha Y.L. (2008). Inhibition of 7,12-dimethylbenz[a]anthracene induced mouse skin carcinogenesis by artemisia capillaris. J. Food Sci..

[42] Yen M.H., Huang C.I., Lee M.S., Cheng Y.P., Hsieh C.J., Chiang L.C., Chang J.S. (2018). Artemisia capillaris inhibited enterovirus 71-induced cell injury by preventing viral internalization. Kaohsiung J. Med. Sci..

[43] Lee J.H., Park E.K., Uhm C.S., Chung M.S., Kim K.H. (2004). Inhibition of helicobacter pylori adhesion to human gastric adenocarcinoma epithelial cells by acidic polysaccharides from artemisia capillaris and panax ginseng. Planta Med..

[44] Yeo D., Hwang S.J., Kim W.J., Youn H.-J., Lee H.-J. (2018). The aqueous extract from artemisia capillaris inhibits acute gastric mucosal injury by inhibition of ros and nf-kb. Biomed. Pharmacother..

[45] Park S.W., Oh T.Y., Kim Y.S., Sim H., Park S.J., Jang E.J., Park J.S., Baik H.W., Hahm K.B. (2008). Artemisia asiatica extracts protect against ethanol-induced injury in gastric mucosa of rats. J. Gastroenterol. Hepatol..

[46] Yang C.-C., Lee M.-R., Hsu S.-L., Chang C.-M.J. (2007). Supercritical fluids extraction of capillarisin from artemisia capillaris and its inhibition of in vitro growth of hepatoma cells. J. Supercrit. Fluids..

[47] Sheu S.J., Chieh C.L., Weng W.C. (2001). Capillary electrophoretic determination of the constituents of artemisiae capillaris herba. J. Chromatogr. A.

[48] Okuno I., Uchida K., Nakamura M., Sakurai K. (1988). Studies on choleretic consituents in artemisia capillaris thunb. Chem. Pharm. Bull..

[49] Wu T.-S., Tsang Z.-J., Wu P.-L., Lin F.-W., Li C.-Y., Teng C.-M., Lee K.-H. (2001). New constituents and antiplatelet aggregation and anti-hiv principles of artemisia capillaris. Bioorg. Med. Chem..

[50] Geng C.A., Huang X.Y., Chen X.L., Ma Y.B., Rong G.Q., Zhao Y., Zhang X.M., Chen J.J. (2015). Three new anti-hbv active constituents from the traditional chinese herb of yin-chen (artemisia scoparia). J. Ethnopharmacol..

[51] Cai H., Song Y.H., Xia W.J., Jin M.W. (2006). Aqueous extract of yin-chen-hao decoction, a traditional chinese prescription, exerts protective effects on concanavalin a-induced hepatitis in mice through inhibition of nf-kappab. J. Pharm. Pharmacol..

[52] Cho H.R., Choi D.H., Ko B.K., Nam C.W., Park K.M., Lee Y.J., Lee S.G., Lee J.S., Lee K.A., Lee E.A. (2000). Cold preservation of rat cultured hepatocytes: The scoparone effect. Transplant. Proc..

[53] Jang S.I., Kim Y.J., Lee W.Y., Kwak K.C., Baek S.H., Kwak G.B., Yun Y.G., Kwon T.O., Chung H.T., Chai K.Y. (2005). Scoparone from artemisia capillaris inhibits the release of inflammatory mediators in raw 264.7 cells upon stimulation cells by interferon-gamma plus lps. Arch. Pharm. Res..

[54] Noh J.R., Kim Y.H., Hwang J.H., Gang G.T., Yeo S.H., Kim K.S., Oh W.K., Ly S.Y., Lee I.K., Lee C.H. (2013). Scoparone inhibits adipocyte differentiation through down-regulation of peroxisome proliferators-activated receptor gamma in 3t3-l1 preadipocytes. Food Chem..

[55] Yang D., Yang J., Shi D., Deng R., Yan B. (2011). Scoparone potentiates transactivation of the bile salt export pump gene and this effect is enhanced by cytochrome p450 metabolism but abolished by a pkc inhibitor. Br. J. Pharmacol..

[56] Zhang A., Sun H., Dou S., Sun W., Wu X., Wang P., Wang X. (2013). Metabolomics study on the hepatoprotective effect of scoparone using ultra-performance liquid chromatography/electrospray ionization quadruple time-of-flight mass spectrometry. Analyst.

[57] Lyu L., Chen J., Wang W., Yan T., Lin J., Gao H., Li H., Lv R., Xu F., Fang L. (2021). Scoparone alleviates ang ii-induced pathological myocardial hypertrophy in mice by inhibiting oxidative stress. J. Cell. Mol. Med..

[58] Fu B., Su Y., Ma X., Mu C., Yu F. (2018). Scoparone attenuates angiotensin ii-induced extracellular matrix remodeling in cardiac fibroblasts. J. Pharmacol. Sci..

[59] Lv H., Sun H., Sun W., Liu L., Wang P., Wang X., Cao H. (2008). Pharmacokinetic studies of a chinese triple herbal drug formula. Phytomedicine.

[60] Yin Q., Sun H., Zhang A., Wang X. (2012). Pharmacokinetics and tissue distribution study of scoparone in rats by ultraperformance liquid-chromatography with tandem high-definition mass spectrometry. Fitoterapia.

[61] Wang Y., Xing X., Cao Y., Zhao L., Sun S., Chen Y., Chai Y., Chen S., Zhu Z. (2018). Development and application of an uhplc-ms/ms method for comparative pharmacokinetic study of eight major bioactive components from yin chen hao tang in normal and acute liver injured rats. Evid. Based Complement. Alternat. Med..

[62] Zhang A., Sun H., Wang X., Jiao G., Yuan Y., Sun W. (2012). Simultaneous in vivo rp-hplc-dad quantification of multiple-component and drug-drug interaction by pharmacokinetics, using 6,7-dimethylesculetin, geniposide and rhein as examples. Biomed. Chromatogr..

[63] Wang X., Lv H., Sun H., Sun W., Liu L., Wang P., Cao H. (2008). Simultaneous determination of 6,7-dimethylesculetin and geniposide in rat plasma and its application to pharmacokinetic studies of yin chen hao tang preparation. Arzneimittelforschung.

[64] Kim J.K., Kim J.Y., Kim H.J., Park K.G., Harris R.A., Cho W.J., Lee J.T., Lee I.K. (2013). Scoparone exerts anti-tumor activity against du145 prostate cancer cells via inhibition of stat3 activity. PLoS ONE.

[65] Choi R.Y., Ham J.R., Lee H.I., Cho H.W., Choi M.S., Park S.K., Lee J., Kim M.J., Seo K.I., Lee M.K. (2017). Scopoletin supplementation ameliorates steatosis and inflammation in diabetic mice. Phytother. Res..

[66] Jang J.H., Park J.E., Han J.S. (2018). Scopoletin inhibits alpha-glucosidase in vitro and alleviates postprandial hyperglycemia in mice with diabetes. Eur. J. Pharmacol..

[67] Nam H., Kim M.M. (2015). Scopoletin has a potential activity for anti-aging via autophagy in human lung fibroblasts. Phytomedicine.

[68] Liu X.L., Zhang L., Fu X.L., Chen K., Qian B.C. (2001). Effect of scopoletin on pc3 cell proliferation and apoptosis. Acta Pharmacol. Sin..

[69] Adams M., Efferth T., Bauer R. (2006). Activity-guided isolation of scopoletin and isoscopoletin, the inhibitory active principles towards ccrf-cem leukaemia cells and multi-drug resistant cem/adr5000 cells, from artemisia argyi. Planta Med..

[70] Seo E.J., Saeed M., Law B.Y., Wu A.G., Kadioglu O., Greten H.J., Efferth T. (2016). Pharmacogenomics of scopoletin in tumor cells. Molecules.

[71] Zeng Y., Li S., Wang X., Gong T., Sun X., Zhang Z. (2015). Validated lc-ms/ms method for the determination of scopoletin in rat plasma and its application to pharmacokinetic studies. Molecules.

[72] Chang Y., Zhang Q.-H., Li J., Zhang L., Guo X., He J., Zhang P., Ma L., Deng Y., Zhang B. (2013). Simultaneous determination of scopoletin, psoralen, bergapten, xanthotoxin, columbianetin acetate, imperatorin, osthole and isoimperatorin in rat plasma by lc–ms/ms for pharmacokinetic studies following oral administration of radix angelicae pubescentis extract. J. Pharm. Biomed. Anal..

[73] Zhao Y.X., Wang M., Dong W.T., Li Y. (2019). Pharmacokinetics, bioavailability and metabolism of scopoletin in dog by ultra-high-performance liquid chromatography combined with linear ion trap-orbitrap tandem mass spectrometry. Biomed. Chromatogr..

[74] Kim J., Lim J., Kang B.Y., Jung K., Choi H.J. (2017). Capillarisin augments anti-oxidative and anti-inflammatory responses by activating nrf2/ho-1 signaling. Neurochem. Int..

[75] Yu Z., Tang L., Chen L., Li J., Wu W., Hu C. (2015). Capillarisin suppresses lipopolysaccharide-induced inflammatory mediators in bv2 microglial cells by suppressing tlr4-mediated nf-kappab and mapks signaling pathway. Neurochem. Res..

[76] Han S., Lee J.H., Kim C., Nam D., Chung W.S., Lee S.G., Ahn K.S., Cho S.K., Cho M., Ahn K.S. (2013). Capillarisin inhibits inos, cox-2 expression, and proinflammatory cytokines in lps-induced raw 264.7 macrophages via the suppression of erk, jnk, and nf-kappab activation. Immunopharmacol. Immunotoxicol..

[77] Kim M., Chun J., Jung H.A., Choi J.S., Kim Y.S. (2017). Capillarisin attenuates exercise-induced muscle damage through mapk and nf-kappab signaling. Phytomedicine.

[78] Tsui K.H., Chang Y.L., Yang P.S., Hou C.P., Lin Y.H., Lin B.W., Feng T.H., Juang H.H. (2018). The inhibitory effects of capillarisin on cell proliferation and invasion of prostate carcinoma cells. Cell Prolif..

[79] Tsui K.H., Chang Y.L., Feng T.H., Hou C.P., Lin Y.H., Yang P.S., Lee B.W., Juang H.H. (2018). Capillarisin blocks prostate-specific antigen expression on activation of androgen receptor in prostate carcinoma cells. Prostate.

[80] Yi Y.-X., Ding Y., Zhang Y., Ma N.-H., Shi F., Kang P., Cai Z.-Z., Zhang T. (2018). Yinchenhao decoction ameliorates alpha-naphthylisothiocyanate induced intrahepatic cholestasis in rats by regulating phase ii metabolic enzymes and transporters. Front. Pharmacol..

[81] Wang X., Sun H., Zhang A., Jiao G., Sun W., Yuan Y. (2011). Pharmacokinetics screening for multi-components absorbed in the rat plasma after oral administration traditional chinese medicine formula yin-chen-hao-tang by ultra performance liquid chromatography-electrospray ionization/quadrupole-time-of-flight mass spectrometry combined with pattern recognition methods. Analyst.

[82] Wang H., Zou H., Ni J., Kong L., Gao S., Guo B. (2000). Fractionation and analysis of artemisia capillaris thunb. By affinity chromatography with human serum albumin as stationary phase. J. Chromatogr. A.

[83] Ogasawara M., Matsubara T., Suzuki H. (2001). Screening of natural compounds for inhibitory activity on colon cancer cell migration. Biol. Pharm. Bull..

[84] Whelan L.C., Ryan M.F. (2004). Effects of the polyacetylene capillin on human tumour cell lines. Anticancer Res..

[85] Masuda Y., Asada K., Satoh R., Takada K., Kitajima J. (2015). Capillin, a major constituent of artemisia capillaris thunb. Flower essential oil, induces apoptosis through the mitochondrial pathway in human leukemia hl-60 cells. Phytomedicine.

[86] Islam M.N., Choi R.J., Jung H.A., Oh S.H., Choi J.S. (2016). Promising anti-diabetic potential of capillin and capillinol isolated from artemisia capillaris. Arch. Pharm. Res..

[87] Lee S.H., Lee J.Y., Kwon Y.I., Jang H.D. (2017). Anti-osteoclastic activity of artemisia capillaris thunb. Extract depends upon attenuation of osteoclast differentiation and bone resorption-associated acidification due to chlorogenic acid, hyperoside, and scoparone. Int. J. Mol. Sci..

[88] Budryn G., Zaczyńska D., Oracz J. (2016). Effect of addition of green coffee extract and nanoencapsulated chlorogenic acids on aroma of different food products. LWT.

[89] Patti A.M., Al-Rasadi K., Katsiki N., Banerjee Y., Nikolic D., Vanella L., Giglio R.V., Giannone V.A., Montalto G., Rizzo M. (2015). Effect of a natural supplement containing curcuma longa, guggul, and chlorogenic acid in patients with metabolic syndrome. Angiology.

[90] Suzuki A., Yamamoto N., Jokura H., Yamamoto M., Fujii A., Tokimitsu I., Saito I. (2006). Chlorogenic acid attenuates hypertension and improves endothelial function in spontaneously hypertensive rats. J. Hypertens..

[91] Ahrens M.J., Thompson D.L. (2013). Effect of emulin on blood glucose in type 2 diabetics. J. Med. Food.

[92] Lee K.-W., Im J.-Y., Woo J.-M., Grosso H., Kim Y.-S., Cristovao A.C., Sonsalla P.K., Schuster D.S., Jalbut M.M., Fernandez J.R. (2013). Neuroprotective and anti-inflammatory properties of a coffee component in the mptp model of parkinson’s disease. Neurotherapeutics.

[93] Shi H., Dong L., Dang X., Liu Y., Jiang J., Wang Y., Lu X., Guo X. (2013). Effect of chlorogenic acid on lps-induced proinflammatory signaling in hepatic stellate cells. Inflamm. Res..

[94] Silva B.A., Ferreres F., Malva J.O., Dias A.C. (2005). Phytochemical and antioxidant characterization of hypericum perforatum alcoholic extracts. Food Chem..

[95] Huang K., Liang X.C., Zhong Y.L., He W.Y., Wang Z. (2015). 5-caffeoylquinic acid decreases diet-induced obesity in rats by modulating pparalpha and lxralpha transcription. J. Sci. Food Agric..

[96] Liang N., Kitts D.D. (2015). Role of chlorogenic acids in controlling oxidative and inflammatory stress conditions. Nutrients.

[97] Yamagata K., Izawa Y., Onodera D., Tagami M. (2018). Chlorogenic acid regulates apoptosis and stem cell marker-related gene expression in a549 human lung cancer cells. Mol. Cell. Biochem..

[98] Liu R., Lai K., Xiao Y., Ren J. (2017). Comparative pharmacokinetics of chlorogenic acid in beagles after oral administrations of single compound, the extracts of lonicera japanica, and the mixture of chlorogenic acid, baicalin, and forsythia suspense. Pharm. Biol..

[99] Gong X., Luan Q., Zhou X., Zhao Y., Zhao C. (2017). Uhplc-esi-ms/ms determination and pharmacokinetics of pinoresinol glucoside and chlorogenic acid in rat plasma after oral administration of eucommia ulmoides oliv extract. Biomed. Chromatogr..

[100] Gu P., Liu R.-J., Cheng M.-L., Wu Y., Zheng L., Liu Y.-J., Ma P.-C., Ding L. (2016). Simultaneous quantification of chlorogenic acid and taurocholic acid in human plasma by lc-ms/ms and its application to a pharmacokinetic study after oral administration of shuanghua baihe tablets. Chin. J. Nat. Med..

[101] Gonthier M.-P., Verny M.-A., Besson C., Rémésy C., Scalbert A. (2003). Chlorogenic acid bioavailability largely depends on its metabolism by the gut microflora in rats. J. Nutr..

[102] Tang D., Li H.J., Chen J., Guo C.W., Li P. (2008). Rapid and simple method for screening of natural antioxidants from chinese herb flos lonicerae japonicae by dpph-hplc-dad-tof/ms. J. Sep. Sci..

[103] Chen J., Mangelinckx S., Ma L., Wang Z., Li W., De Kimpe N. (2014). Caffeoylquinic acid derivatives isolated from the aerial parts of gynura divaricata and their yeast alpha-glucosidase and ptp1b inhibitory activity. Fitoterapia.

[104] Hao B.J., Wu Y.H., Wang J.G., Hu S.Q., Keil D.J., Hu H.J., Lou J.D., Zhao Y. (2012). Hepatoprotective and antiviral properties of isochlorogenic acid a from laggera alata against hepatitis b virus infection. J. Ethnopharmacol..

[105] Cao Z., Ding Y., Cao L., Ding G., Wang Z., Xiao W. (2017). Isochlorogenic acid c prevents enterovirus 71 infection via modulating redox homeostasis of glutathione. Sci. Rep..

[106] Liu X., Huang K., Niu Z., Mei D., Zhang B. (2019). Protective effect of isochlorogenic acid b on liver fibrosis in non-alcoholic steatohepatitis of mice. Basic Clin. Pharmacol. Toxicol..

[107] Tian Y., Li Q., Zhou X., Pang Q., Xu Y. (2017). A uhplc-ms/ms method for simultaneous determination of twelve constituents from erigeron breviscapus extract in rat plasma: Application to a pharmacokinetic study. J. Chromatogr. B Analyt. Technol. Biomed. Life Sci..

[108] Huang L.H., Xiong X.H., Zhong Y.M., Cen M.F., Cheng X.G., Wang G.X., Zang L.Q., Wang S.J. (2016). Pharmacokinetics of isochlorgenic acid c in rats by hplc-ms: Absolute bioavailability and dose proportionality. J. Ethnopharmacol..

[109] Haq F.U., Roman M., Ahmad K., Rahman S.U., Shah S.M.A., Suleman N., Ullah S., Ahmad I., Ullah W. (2020). Artemisia annua: Trials are needed for covid-19. Phytother. Res..

[110] Zhang X., Zhao Y., Guo L., Qiu Z., Huang L., Qu X. (2017). Differences in chemical constituents of artemisia annua l from different geographical regions in china. PLoS ONE.

[111] Lang S.J., Schmiech M., Hafner S., Paetz C., Steinborn C., Huber R., Gaafary M.E., Werner K., Schmidt C.Q., Syrovets T. (2019). Antitumor activity of an artemisia annua herbal preparation and identification of active ingredients. Phytomedicine.

[112] Tsai K.C., Huang Y.C., Liaw C.C., Tsai C.I., Chiou C.T., Lin C.J., Wei W.C., Lin S.J., Tseng Y.H., Yeh K.M. (2021). A traditional chinese medicine formula nricm101 to target covid-19 through multiple pathways: A bedside-to-bench study. Biomed. Pharmacother..

